# Unsymmetrical zinc phthalocyanines containing thiophene and amine groups as donor for bulk heterojunction solar cells

**DOI:** 10.3906/kim-2010-1

**Published:** 2021-06-30

**Authors:** Gülnur KESER KARAOĞLAN, Öznur DÜLGER KUTLU, Ahmet ALTINDAL

**Affiliations:** 1 Department of Chemistry, Faculty of Arts and Sciences, Yıldız Technical University, İstanbul Turkey; 2 Department of Physics, Faculty of Arts and Sciences, Yıldız Technical University, İstanbul Turkey

**Keywords:** Unsymmetrical phthalocyanine, amine, thiophene, bulk heterojunction solar cell, photovoltaic

## Abstract

Photovoltaic technology is an alternative resource for renewable and sustainable energy and low costs organic photovoltaic devices such as bulk-heterojunction (BHJ) solar cells, which are selective candidates for the effective conversion of solar energy into electricity. Asymmetric phthalocyanines containing electron acceptor and donor groups create high photovoltaic conversion efficiency in dye sensitized solar cells. In this study, a new unsymmetrical zinc phthalocyanine was designed and synthesized including thiophene and amine groups at peripherally positions for BHJ solar cell. The structure of the targeted compound (**4**) was characterized comprehensively by FT-IR, UV–Vis, ^1^H-NMR, and MALDI-TOF MS spectroscopies. The potential of this compound in bulk heterojunction (BHJ) photovoltaic devices as donor was also researched as function of blend ratio (blend ratio was varied from 0.5 to **4**). For this purpose, a series of BHJ devices with the structure of fluorine doped indium tin oxide (FTO)/poly(3,4-ethylenedioxythiophene):poly(styrene sulfonate)/ ZnPc:[6,6]- phenyl-C61- butyric acid methyl ester (PCBM) blend/Al with identical thickness of ZnPc:PCBM layer were fabricated and characterized. Photo current measurements in **4** revealed that the observed photo current maximum is consistent with UV-vis spectra of the compound of **4**. Preliminary studies showed that the blend ratio has a critical effect on the BHJ device performance parameters. Photovoltaic conversion efficiency of 6.14% was achieved with **4** based BHJ device.

## 1. Introduction

Due to the negative impact on the environmental using fossil fuels, the investigation for renewable energy research has taken its place as an indispensable subject of study worldwide. Therefore, photovoltaic organic solar cells (OSCs) based on organic sources have been the center of rising attention due to their flexibility, light weight, solution processability and as an inexpensive cost photovoltaic energy material [1–3]. The efficiency of photovoltaic organic solar cells is greatly increased by adding of the bulk-heterojunction (BHJ) term [4–8], an active fragment where electron acceptor and donor materials are blended in a solution and placed into a thin film sandwiched between two electrodes. Recently, huge progresses are being accomplished in the development of the power conversion capacity (PCE) of organic bulk-heterojunction solar cells. BHJ solar cells, based on polymer / fullerene combination, have attracted a great deal due to power conversion efficiency of over 10% [9–13]. Higher power conversion efficiencies are now obtained using low-band gap polymers that allow the collection of a wider segment of the solar spectrum [14,15].

Material innovation is an important factor that determines the efficiency of organic solar cells. Some polymer types have been preferred as electron donor in solar cell studies [16]. Fullerenes and their derivatives, which have been widely preferred as electron acceptor materials in OSCs and the poly(3-hexylthiophene-2,5-diyl) (P3HT) as the electron donor, are among the most widely used materials for industrialization in BHJ solar cell technology [17]. [6,6]-phenyl-C61- butyric acid methyl ester (PCBM), one of these fullerene derivatives, has fabulous photovoltaic features [18].

Phthalocyanines (Pcs), which are decent p-type semiconductors, offer active redox chemistry that can be modulated as a function of the periferal substitute groups and / or the central metal in the aromatic space of the pc ring. Consequently, when photoexcited, Pcs are capable of acting either as lectron-acceptors when linked to donor systems such as polythiophenes [19] or electron-donors when they are connected to suitable electron-acceptor groups such as fullerenes [20]. Although significant progress has been made for high-performance BHJ solar cells with P3HT, it has some disadvantages, such as a restricted absorption wavelength [21]; hence, there is an obligation to improve new donor materials having larger absorption in the red region. For this purpose, Pcs are commonly used as donor compounds in solar cells. All these properties make these structures worthy photoactive materials. Normally, P3HT molecule absorbs light from 400 to 600 nm, but if it is connected to a Pc molecule absorbs light at wavelengths between 600 and 700 nm to the active layer will lead to a broadening of the absorption range. Lately, the clear contribution of the Pc around 700 nm to the photocurrent has been proved by Torres et al [22]. There are many studies on thiophene derivatives as π-conjugated organic molecules because of their structural planarity permits strong electronic conjugation within the structure as well as their stability and well-known synthetic chemistry. For these reasons, attachment of these thienyl motions at the Pc ring as peripherally positions might result in the enlargement and improvement of the π-conjugation systems [23,24].

We report herein that the synthesis of unsymmetrical tetra substituted zinc phthalocyanines linked thiophene and amine groups at the peripheral positions and the use of electron donating novel phthalocyanines obtained in BHJ devices as an alternative to P3HT material has yielded successful results. The thiophene groups were chosen to provide the electron-releasing effect for the electronic properties of phthalocyanines. The combination of the sulfur atom in thiophene and amine groups as substituent in newly designed unsymmetrical zinc phthalocyanine has significantly improved performance of BHJ.

## 2. Experimental design 

All information about the used materials, equipment, synthesis, improved performance of BHJ, and photovoltaic behaviours were shown in the “Supplementary Information” Section [25,29].

## 3. Results and discussion

### 3.1. Synthesis and characterization

**Figure 1:**
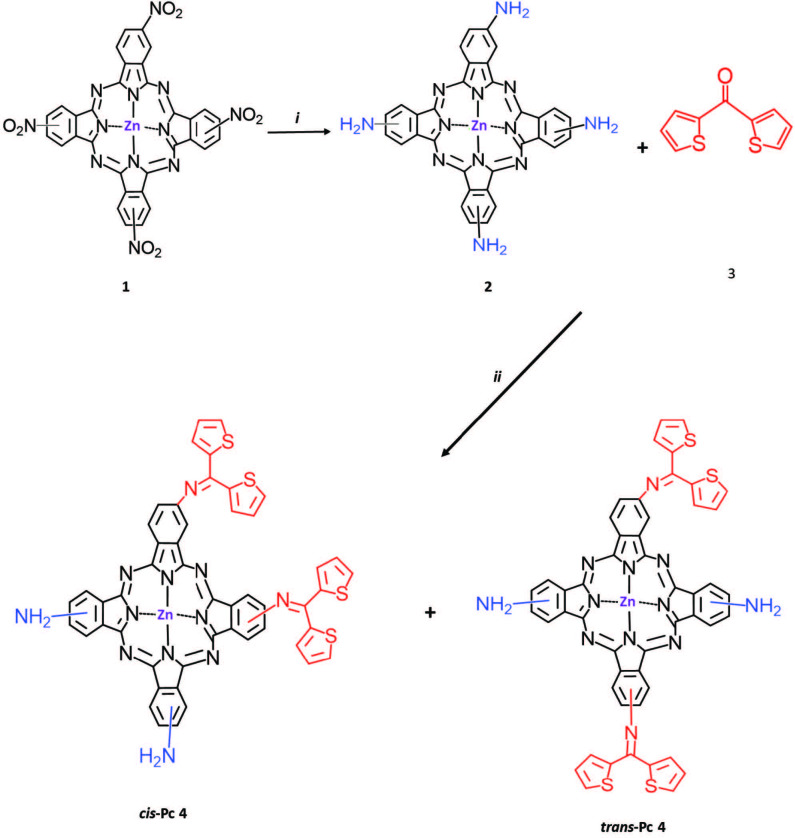
Synthetic route of Pc **4**: (i) Pd/C, hydrazine hydrate, 1,4-dioxane; (ii) dry DMF, *p*-TsOH H_2_O, 48 h, reflux.

Today, asymmetric metal phthalocyanines (MPcs) have been the focus of attention in order to fine-tune the properties of these complexes because symmetrical MPcs do not always meet the requirement for developing large technology applications [30]. The new unsymmetrical phthalocyanine including thiophene groups ( **4** ) was synthesized step by step. After tetra-nitro-ZnPc derivative ( **1** ) was synthesized, tetra-amine-ZnPc ( **2** ) was synthesized by the reduction of tetra-nitro-ZnPc in the medium of hydrazine hydrate and 10% Pd/C as the catalyst [25]. Finally, **4** was obtained with the statistical reaction of ( **2** and **3** ) affording in principle a mixture of two A_2_B_2_ type unsymmetrical zinc phthalocyanines complexes (Figure 1).

After chromatographic separation, unique spot was obtained for complex **4** . To approve chemical structure of the new asymmetric Pc complex ( **4** ), spectroscopic methods were used such as UV–Vis, FT-IR, ^1^H-NMR and MALDI-TOF MS spectroscopies. Most of the phthalocyanines, especially unsubstituted ones, have low solubility in many organic solvents; however, substation of suitable functional groups on the Pc ring improves the solubility of some chemicals such as thiophene, alkyl, phenoxy and alkoxy groups. Pc ( **4** ), which is obtained by binding thiophene groups to tetra amine Pc ( **2** ), showed good solubility like Pc ( **2** ) in many common solvents such as THF, CHCl_3_, DMF, and DMSO. 

For unsymmetrical zinc phthalocyanine derivation ( **4** ), the characteristic -N=C stretch at 1679 cm^–1^ was appeared in the FTIR spectrum, indicative of expected structure. The characteristics vibrations corresponding to amine groups (-NH_2_) were observed at 3343, 3231, 1609 cm^–1^ (for **4** ). Aromatic CH stretching at 3064 cm^–1^ was observed for the complex. Stretching vibration of thiophene rings in Pc complex **4** was also detected at 833 cm^−1^.

**Figure 2:**
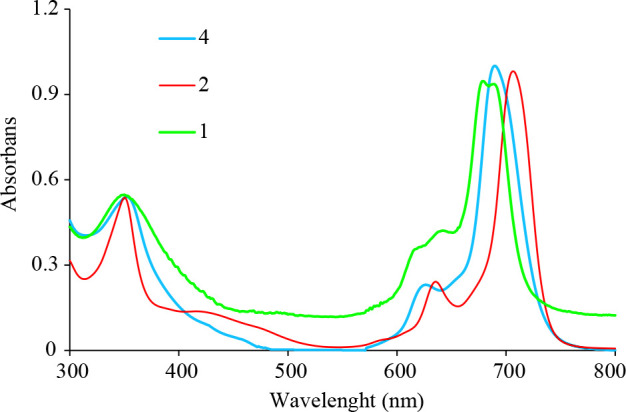
UV-Vis absorption spectra of complex 1, 2 and 4 in THF (1.0   10^-5^ M).

The UV–Vis spectra of the Pcs **1** , **2** and **4** are given in Figure 2 in THF. In these spectra, two bands were observed: Soret or B band (350–352 nm) in the UV region and Q band (679–707 nm) in the visible region [31]. When the UV-Vis spectra of compounds **1** and **2** are compared, the shift observed between the Q band absorption peaks is caused by the reduction of the -NO_2_ groups in the peripheral positions to the electron donor -NH_2_ groups. Spectra of **2** and **4** in THF have intense Q bands at 707 and 690 nm due to a single π- π* transition with shoulders at 635 and 626 nm, respectively. Nine nm blue shifting was observed for asymmetric Pc compared to amine Pc due to binding thiophene groups on peripherally positions. However, the Soret (B) bands are observed at similar wavelengths as 350 and 352 nm, respectively (Table 1).

**Table 1:** UV-Vis data for zinc phthalocyanine complexes (1, 2 and 4) in THF.

Complex	λmax, nm (log ε, L mol-1 cm-1)
1 350 (4.73) 617a (4.55) 639a (4.62) 679 (4.97) 689 (4.97)	
2 350 (4.73)	635a (4.38) 707 (4.99)
4 352 (4.73)	626a (4.36) 690 (4.99)
a Shoulder	

The newly synthesized Pc **4** was characterized by ^1^H-NMR spectrum, which was observed to be in good correlation with its own structure. **4** showed the phthalocyanine skeleton protons and thiophene protons as multiples between 9.06 and 6.99 ppm as expected. The -NH_2_ protons at 4.38 ppm as singlet for 4 protons.

Also unsymmetrical Pc **4** was characterized by MALDI-TOF MS where molecular ion peak atm/z: 687.437 [M-2NH_2_ –C_9_H_6_NS_2_-C_4_H_3_S+2H]^+^, 1013.27 [M+Na+2H]^+^., clearly indicates the formation of desired products as A_2_B_2_ types. 

In the Figure 1, it is seen that the spectral data of the newly synthesized compounds confirm the proposed structures.

### 3.2. Photovoltaic characterization

**Figure 3:**
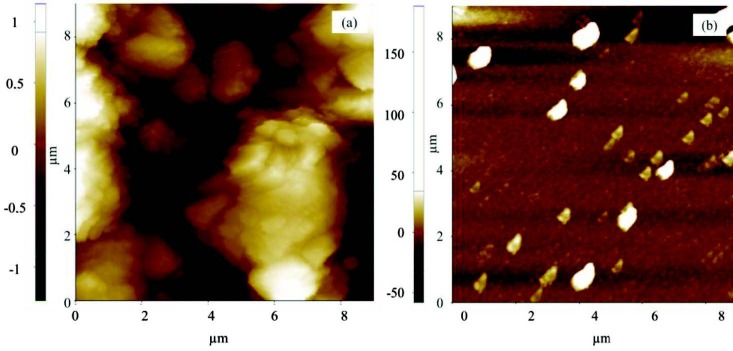
AFM topography of the films with 4.0:1.0 (a) and 2.5:1.0 (b) blend ratios.

It is well known that the surface morphology of the blend film is also important for the photovoltaic performance of the phtalocyanine based bulk heterojunction solar cells. In order to clarify the effect of the surface morphology of the blend film on the photovoltaic performance, the surface morphology of the blend film was analyzed by atomic force microscopy. The AFM image analysis was performed with commercial programmes associated with XEI and XEP software application to determine the surface roughness characterized by the root mean square (RMS) parameter. As a represantative results, Figures 3 (a) and 3 (b) shows the AFM surface topography of the blend films of **4.0:** 1.0 and **2.5:** 1.0, respectively. It is clear that the films’ morphology were considerably affected by the **4** ratio. The film with **4.0** :1.0 blend ratio exhibited higher surface roughness, while films with **2.5** :1.0 blend ratio have displayed lower root mean square (rms) roughness. From the close analysis of the AFM images, the surface roughness was determined and was found to be 60 and 45 nm for the film with **4.0** :1.0 and **2.5** :1.0 blend ratio, respectively. 

Before the photo voltaic characterization, the conductivity of the PEDOT:PSS film and the photocurrent vs. incident light wavelength measurements were carried out. Our results indicated that the conductivity of the PEDOT:PSS film was about 0.35 S/cm. 

**Figure 4:**
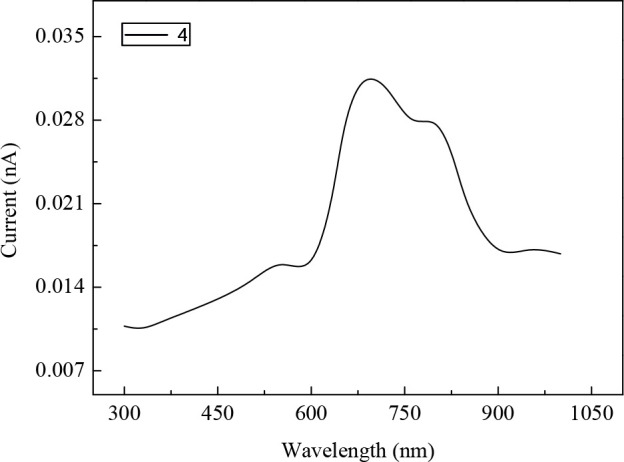
Variation of the photocurrent in 4 with the incident light wavelength.

The variation of the photo current with the incident light wavelength is presented in Figure 4 for the film of **4** . It should be mentioned here that maximum photoconductivity was obdserved with **4** based film for all incident light wavelength. As is clear, the photo current increases with the increase in incident light wavelength, goes through a maximum at a certain wavelength and then decreases. 

Maximum photocurrent was observed at about 690 nm which is consistent with UV-Vis spectra of the compound of **4** . The wavelength dependence of the monitored photo current expresses that vigorously absorbed photons are mainly responsible for free carrier production and, therefore, photocurrent.

**Figure 5:**
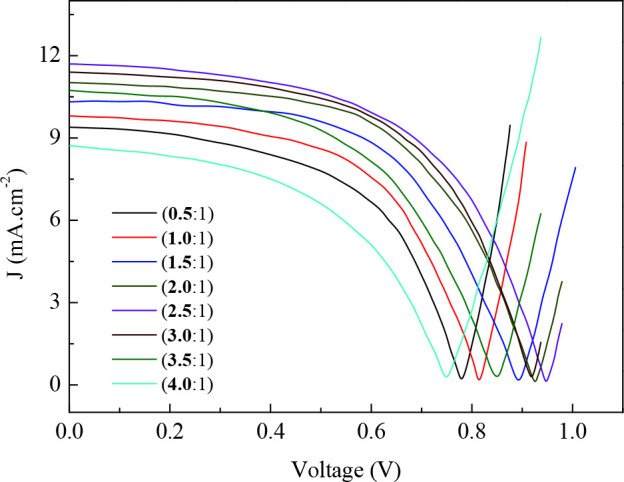
J-V characteristics of the 4 based device with various blend ratio.

In order to evaluate the photovoltaic performance of **4** in BHJ solar cells as donor and the effect of the blend ratio on the main performance parameters, a series of BHJ devices with FTO/PEDOT:PSS/ **4** :PCBM blend/Al structure were fabricated and characterized. During the fabrication and characterization studies, the ratio of PCBM was fixed at 1, and the ratio of donor ( **4)** was varied from 0.5 to 4.0 because of its well-known critical impact on the device performance [32]. Current density-voltage (J-V) characteristics obtained with the FTO/PEDOT:PSS/ **4** :PCBM blend/Al structure of BHJ solar cells devices with various blend ratio under AM 1.5 G illumination is shown in Figure 5 and evaluated performance parameters tabulated in Table 2. At first glance, it is obvious that all the devices fabricated exhibit rectifying behavior with various rectification ratios depending on the blend ratio. It is also clear that the photovoltaic performance parameters of the devices are modulated by the blend ratio of **4** : PCBM. As usual, photovoltaic conversion efficiency of a solar cell which is defined a

**Table 2:** Blend ratio dependence of the photovoltaic parameters for the investigated devices.

Blend Ratio	VOC((V)	J JSC(mA cm–2)	Vm(V)	Jm(mA cm–2)	FF	h(%)
(0.5:1.0)	0.78	9.04	0.57	7.02	0.57	4.01
(1.0:1.0)	0.81	9.80	0.59	7.76	0.58	4.57
(1.5:1.0)	0.89	10.31	0.64	8.28	0.58	5.30
(2.0:1.0)	0.93	11.02	0.62	9.30	0.56	5.77
(2.5:1.0)	0.95	11.70	0.68	9.03	0.55	6.14
(3.0:1.0)	0.92	11.40	0.66	9.10	0.57	6.00
(3.5:1.0)	0.85	10.7 4	0.62	7.84	0.53	4.84
(4.0:1.0)	0.75	8.73	0.51	6.51	0.51	3.32

(1)η=Pm/Pin

where *P* *_in_* is the power of incident light, *P* *_m_* is maximum power output of the cell, which is defined as

(2)Pm=JSCxVOCxFF

in the following equation; FF expressed as the filling factor is defined as the maximum power output of the solar cell per unit area divided by the product of *V* *_OC_* and *J* *_SC_* . 

(3)FF=(JmxVm)/(JSCxVOC)

where *J* *_m_* and *V* *_m_* are the current density and voltage at which the cell delivers the maximum power density. Interestingly, it was observed that the open circuit voltage ( *V* *_OC_* ) of the devices increases with the increase of **4** ratio in the blend solution; when the blend ratio of **4** :PCBM reached **2.5** :1, the best **4** :PCBM based cell was realized as shown in Figure 5, and then *V* *_OC_* decreases with further increase of **4** ratio (see Table 2).

From the close investigation of the Table 2, it will be clear that the performance of the devices strongly depends on the blend ratio, photovoltaic conversion efficiency of the device varies between 3.32% for **4.0** :1.0 blend ratio and 6.14% for **2.5** :1.0 blend ratio. The *V* *_OC_* , *J* *_SC_* , FF, and efficiency of the champion device were 0.95 V, 11.70 mA/cm^2^, 0.55, and 6.14 %, respectively. To the best of our knowledge, these values are the highest reported for phthalocyanine based bulk heterojunction solar cells. In order to be sure that the dependence of the observed open circuit voltage on blend ratio is repeatable, the J-V measurements were repeated for another set of the devices from the same batch of the devices and these measurements verified that the observed dependence of open circuit voltage on the blend ratio are reproducible, except for a small shift in its value. It is well known that P3HT and PCBM are frequently preferred donor and acceptor substances in BHJ devices. Previous works on the BHJ devices made from P3HT: PCBM blends with various blend ratios have indicated that the photo voltaic performances of these devices strongly depend on the blend ratio [33–35].

The energetic disturbance of organic semiconductors, compared to their crystalline inorganic counterparts, causes the intramolecular and intermolecular interactions in a morphologically diverse film to expand the distribution of electronic states and is significantly affected by structural properties. Regardless of the functional shape (Gaussian or an exponential function-or a combination of these two) of density of states, increased broadening of the density of states invariably pushes tail states further into the band gap, and this leads to strong correlations between disorder and voltage losses in solar cells [36]. More recent reports on planar heterojunction [37] as well as bulk heterojunction solar cells [38–40] have shown that the open circuit voltage is strongly dependent on the difference between the highest occupied molecular orbital (HOMO) of the donor and the lowest unoccupied molecular orbital (LUMO) of the acceptor materials. It is proposed that the nanoscale morphology of the two components (donor/acceptor) in the photoactive layer and the efficient separation of charges at the donor– acceptor interface in bilayer planar and non-planar metal Pc/ C60 solar cells are also crucial in determining the *V* *_OC_* value. Relationship between energetic disorder and open-circuit voltage in bulk heterojunction organic solar cells has been investigated by Blakesley et al [41]. They were reported that the open circuit voltage associated with the charge-carrier recombination rates, donor-acceptor energy gap, contact work functions, illumination intensity, and the amount of energetic disorder. The complex factors leading open circuit voltage losses through energetic disorder in BHJ solar cells have been investigated by Nguyen et al [42]. It was reported that disorders contribute as much as 0.2 V of *V* *_OC_* loss. A reasonable explanation for the observed ratio dependence has been given by the eutectic phase behavior of donor and acceptor blends and suggested that morphology at the optimum composition ratio is slightly hypoeutectic [43,44]. 

To access efficient exciton dissociation in BHJ devices, the randomly oriented donor acceptor interfaces are employed, and the performance of the devices are determined by the morphology of the donor-acceptor interface [45,46], the molecular orientation, and aggregation behavior [47,48]. On the other hand, it is well known that interfacial energetic, which involves tightly bounded singlet excitonic states and loosely bounded charge transfer states, has direct impacts on open circuit voltage of the BHJ devices [46,49,50] It was reported previously that the upper limit of the open circuit voltage is determined by the loosely bounded charge transfer states and their disordered effect [51–53]. The obtained J−V characteristics for devices with a fixed donor material and different fullerene-based acceptor materials indicated that, as the acceptor with higher lowest unoccupied molecular orbital level is employed, the open circuit voltage becomes larger due to the increased effective band gap [54]. 

Metal-insulator-metal (MIM) model is widely used to interpret and analyze the obtained open circuit voltage data. The MIM model assumes that the upper limit of the *V* *_OC_* is determined by the work function difference of the anode and cathode materials. Figure 5 clearly shows that, if the origin of the *V* *_OC_* is due to the work function difference all the devices should yield a *V* *_OC_* values of 0.47 (Work functions of FTO and Al is 4.80 and 4.33 eV, respectively), the MIM model can not be applied to our devices. More recently, many theoretical and experimental studies on BHJ have shown that the *V* *_OC_* is independent of the choice of cathode materials and depends on various factors including, energetic disorder in active layer, donor-acceptor energy gap, and rate of charge recombination [55–57]. Effects of blend composition on the morphology of Si-PCPDTBT: PC71BM based bulk heterojunction organic solar cells have been studied by Lin et al. [58], and it was reported that the exciton dissociation efficiency is highly dependent on the content of blend. For further understanding of the composition dependence of the performance of a BHJ device, the structural evolution during blend crystallization for P3HT:PCBM blends were investigated by Barrena et. al. [59]. They reported that donor:acceptor blends with ratios of 1.0:0.5, 1.0:0.8, and 1.0:2.0 exhibit differing microstructure during solidification. Therefore, the observed blend ratio dependence for open circuit voltage can be related to a differing nature of the microstructure of the blend films.

High level blend ratio of **4** give rise to more disordered film formation because of the high aggregation tendency of phthalocyanine molecule. Increased disordering in active layer leads to a decrease in exciton diffusion length, which destabilizes the charge separated states. Destabilization of the charge separated states leads to an increase in charge recombination rate, which could result in a decrease in *V* *_OC_* . The observed trend for short circuit current density supports this conclusion. By a close analysis of the Figure 5, it becomes clear that the short circuit current density follows the same trend with VOC, it increases with the increase of **4** ratio in the blend solution and reaches a maximum when the blend ratio of **4** :PCBM reached **2.5** :1. As it is well known, short circuit current density is another key parameter for a solar cell, which is determined by the product of photoinduced charges and their mobility. It was reported before by Gulbinas et al. [57] that the short circuit current density in a BHJ solar cell device is determined by the charge separation into free carriers which is strongly influenced by the blend ratio. It was also reported that the charge separation is efficient in PCBM rich blends, suggesting that high mobility of one type of carriers is essential for efficient charge separation, and morphology optimization doubles the charge pair separation efficiency and the short circuit current density. It can be again concluded that the structural evaluation during blend crystallization for **4** :PCBM blends play crucial role in defining the basic performance parameters of a BHJ device.

## 4. Conclusion

Unsymmetrical zinc phthalocyanine ( **4** ) bearing thiophene and amine groups as donor was successfully synthesized as confirmed by FT-IR, UV-Vis, ^1^H-NMR, and MALDI-TOF MS. Bulk heterojunction solar cell devices using blended **4** :PCBM with eight different donors: acceptor ratios have been fabricated and characterized. Our preliminary results showed that the entire device fabricated exhibited photovoltaic character. It was also found that the ratio of donor: acceptor has a significant effect on the photovoltaic behavior of the devices. Photovoltaic conversion efficiency of 6.14% was achieved with a **4** based device.
